# Associations of physical activity and screen time with suboptimal health status and sleep quality among Chinese college freshmen: A cross-sectional study

**DOI:** 10.1371/journal.pone.0239429

**Published:** 2020-09-18

**Authors:** Chenjin Ma, Long Zhou, Wangli Xu, Shuangge Ma, Yu Wang

**Affiliations:** 1 Center for Applied Statistics and School of Statistics, Renmin University of China, Beijing, China; 2 School of Public Health, Yale University, New Haven, Connecticut, United States of America; National University of Singapore, SINGAPORE

## Abstract

This study aimed to investigate the associations of physical activity (PA) and screen time (ST) with physiological, psychological, and social health—particularly regarding effects on sleep quality—among Chinese college freshmen. A cross-sectional survey was conducted at Renmin University of China, in Beijing. A total of 5,233 students were surveyed in September 2015. Participants completed a self-report questionnaire on their demographic characteristics, tobacco and alcohol use, PA, ST, sleep quality, and health status. Multivariate logistic regression was performed to examine the independent and interactive associations between PA and ST with sleep quality and suboptimal health status. In total, 10.43%, 13.18%, and 13.26% of the 5,233 students had physiological, psychological, and social suboptimal health status, respectively. The prevalence of poor sleep quality was 37.94%. High ST and high PA were significantly associated with physiological suboptimal health status (aOR = 1.39, 95% CI: 1.16–1.68, and aOR = 0.55, 95% CI: 0.45–0.71), psychological suboptimal health status (aOR = 1.43, 95% CI: 1.21–1.69, and aOR = 0.57, 95% CI: 0.47–0.69), social suboptimal health status (aOR = 1.27, 95% CI: 1.08–1.50, and aOR = 0.63, 95% CI: 0.52–0.77), and poor sleep quality (aOR = 1.20, 95% CI: 1.03–1.39, and aOR = 0.64, 95% CI: 0.55–0.76). Additionally, low ST and high PA were interactively negatively associated with poor sleep quality (aOR = 0.56, 95% CI: 0.45–0.70), physiological suboptimal health status (aOR = 0.49, 95% CI: 0.40–0.59), psychological suboptimal health status (aOR = 0.48, 95% CI: 0.39–0.58), and social suboptimal health status (aOR = 0.49, 95% CI: 0.40–0.59). These findings suggested there are independent and interactive associations of low ST and high PA with poor sleep quality and suboptimal health status among Chinese college freshmen.

## Introduction

In a period of great change and social-developmental transition, many studies focus on college students [1–3], who are generally considered to be relatively healthy; however, researchers have increasingly suggested they can have various physiological, psychological, and social health problems [4–7]. In the physical domain, traditional physical health refers to the state where the individual has no disease and each physiological index is normal [8, 9]. In the psychological domain, one can show the capacity to cope and recover from psychological stress and prevent post-traumatic stress disorders [10]. Social health requires not only healthy individuals but also a healthy social environment, which includes social adjustment, resources, and support [11].

Specifically, studies monitoring physical health have found that, since 2010, Chinese college students have demonstrated an overall decline in physical indicators [12], such as speed, strength, and endurance [13]. Some studies have further indicated that college students worldwide have experienced increased levels of depression [4, 6, 7]. In American colleges, the percentage of students diagnosed with depression increased from 10% in 2000 to 15% in 2006 [14]. A study in Beijing, China found that 33.6% of 1,186 university students showed depressive symptoms [5]. Online data from 33,943 students at 39 colleges in California indicated that 19% had experienced psychological distress in the past 30 days [1]. Additionally, it has been reported that, among college students, there is a high prevalence of poor sleep quality, with 52.7% classified as having a poor sleep quality [15]. Such a finding is concerning, as sleep quality has been shown to have significant negatively effects on academic performance and test scores [16]. Furthermore, college students with poor sleep quality may suffer from headaches, insomnia, fatigue, and memory problems [17]. Thus, sleep quality has direct and indirect effects on college students’ academic function, physical, psychological health, quality of social relationships, social support [18, 19]. Promoting sleep quality is important to the prevention of health problems.

Physical inactivity and screen-based sedentary behaviors are serious public health concerns faced by young adults in many countries [20–22]. The benefits of high PA and low ST on physical health are well studies and documented, such as lower blood cholesterol, lower blood pressure, reduced obesity, higher bone density, vision and eye health [23, 24]. Some studies have investigated the relationships among physical activity (PA), screen time (ST), and sleep quality, and how they relate to psychological health [25–27]; however, findings have been inconsistent. Two studies, conducted in the United Stated and England, failed to find an association between PA and psychological health [28, 29], while studies from China reported that high ST and low PA have adverse effects on sleep quality and psychological health among college students [30, 31]. Some studies in China also showed that high ST and low PA can interact to cause psychological problems [32, 33]. Furthermore, a literature review suggested that independent associations of PA and ST with social health have received very little investigation [34], and less is known as to whether PA and ST interact and have an additive or compensatory effect on social health.

Thus, we aimed to investigate the independent and interactive associations of ST and PA with physiological, psychological, and social health, as well as sleep quality, among Chinese college freshmen. Based on the significant results, it will help filling current gaps in the literature.

## Materials and methods

### Sample and data collection

This study was conducted in September, 2015 at the Renmin University of China (RUC), a large public university in Beijing. All participants received a self-report questionnaire during their physical examination at the RUC school hospital. This was a routine procedure for all new students, including bachelor’s, master’s, and doctoral students. A total of 6,025 questionnaires were distributed, and 5,233 completed responses were received (792 not return the questionnaires), with a response rate of 86.5%. For the missing, the number of each variable may be different (age: 106; student type: 14; residential background: 45; weight loss: 29; usage of tobacco: 4; usage of alcohol: 5; ST: 23; PA: 41). Before completing the survey, each student underwent a health examination at the hospital. The examination provided information on medical history, a physical examination, blood hematology and biochemistry analysis, a resting electrocardiogram, and chest radiography. Students with abnormal results were required to be re-examined. A total of 111 students (2.1%) were diagnosed with a disease that can be found in ICD-9 codes (a list of codes for International Statistical Classification of Diseases and Related Health Problems). Data on those students were removed from analysis.

The questionnaire consisted of four sections: student characteristics, health-related behaviors, sleep quality evaluation, and the Sub-Health Measurement Scale V1.0 (SHMS V1.0). The questionnaire is presented in the Supplementary Materials (S1 File). Student characteristics included gender, age, student type (bachelor’s, master’s, or doctoral student), and residential background (rural or urban). Age was divided into three groups (<20, 20–25 and >25 years old).

Assessed health-related behaviors included alcohol use, tobacco use, PA, ST, and weight loss over the past six months. Alcohol use was indicated by a single question “Do you drink alcohol in the past six months?” Tobacco use was indicated by a single question “Do you smoke in the past six months?” Weight loss was indicated by the question “Do you try to lost weight in the past six months?” Alcohol use, tobacco use, and weight loss were measured using binary variables, with “0” representing no such behavior and “1” representing having this behavior in the past six months. PA was assessed by days per week (0–7 days) that a student participated in sports such as running, swimming, playing ball, etc. Students with high PA levels were those who participated in sports at least three days per week. Such dichotomy has been previously used in recognized student surveys and has been shown to produce valid and reliable responses among school-age youth [35–37], as well as college students [30, 31]. Therefore, referring to their approach, the dichotomy was also used in this study to measure PA. The overall ST was assessed using the following question: “During the past six months, how many hours per day did you spend on a computer, including Internet use, watching TV or video programs, and playing games, as well as using other kinds of electronic equipment, on average?” Following some published studies [35–37], we categorized ST as low (≤ 2 h/day) and high (> 2 h/day).

Sleep quality was measured using the Pittsburgh Sleep Quality Index (PSQI). The PSQI is a self-rating questionnaire and consists of seven components: overall sleep quality, duration of sleep, sleep latency, sleep efficiency, sleep disturbances, use of sleep medications, and daytime dysfunction [38]. The overall score is summed from these seven components, with higher scores indicating poorer sleep quality. Its good reliability and validity could be shown in some studies [39, 40]. The form of PSQI is presented in the Supplementary Materials (S2 File). According to published literature, an overall score > 5 can be defined as “poor sleep quality” [3].

Participants’ health, including physiological, psychological, and social health, was assessed using the SHMS V1.0, which is a multidimensional, self-report symptom inventory developed by a research group in China [32]. This standardized questionnaire has been widely applied in some health-related studies [33, 41, 42]. The reliability of SHMS V1.0 has been confirmed with a Cronbach’s α of 0.679 in this study. The SHMS V1.0 consists of 39 items, 35 of which are divided among three symptom dimensions: physiological symptoms (14 items), psychological symptoms (12 items), and social symptoms (nine items). The remaining four items are health self-evaluations. Physiological symptoms comprise the following factors: physical condition (three items), organ function (six items), body movement function (three items), and vigor (two items). Psychological symptoms consist of three parts: positive emotions (four items), psychological symptoms (six items), and cognitive function (two items). Social symptoms consist of three parts: social adjustment (four items), social resources (three items), and social support (two items). For each item, there are five response categories: 1 = never, 2 = occasionally, 3 = sometimes, 4 = constantly, and 5 = always. Participants were asked to report uncomfortable symptoms they experienced during the past six months. Total scores in dimension were calculated; the original score of each factor was equal to the total score of items included in that factor, and the original score of each dimension was equal to the total score of included factors. Then, the original score of each dimension was converted to obtain the final score using the following formula:

$$\text{converted}\;\text{score}\;\text{in}\;\text{dimension}=\frac{\text{original}\;\text{total}\;\text{score}\;\text{in}\;\text{dimension}-\text{theroretically}\;\text{lowest}\;\text{score}\;\text{in}\;\text{dimension}}{\text{theroretically}\;\text{highest}\;\text{score}\;\text{in}\;\text{dimension}-\text{theroretically}\;\text{lowest}\;\text{score}\;\text{in}\;\text{dimension}}\times 100\%$$ (1)

The converted score, which was the total score of health status and ranged from 0 to 100, was used in downstream analysis. A lower total score corresponded to a worse health status.

As such, health status in our analysis consisted of two categories: healthy and having suboptimal health. With the unilateral P10 point of all dimensions of the cohort as the criterion, the cutoffs for physiological, psychological, and social suboptimal health were 66.1, 52.1, and 55.6, respectively. When the score for these three dimensions was lower than the cutoff, participants were considered to have physiological, psychological, and/or social suboptimal health. If a student did not have SHS with respect to any of these three dimensions, he or she was considered healthy.

### Statistical analysis

Analyses were conducted using SAS 9.4. Continuous variables with distributions close to normal were summarized using means and standard deviations. Those with distributions deviating from normal were summarized using medians and quartiles, and categorical variables were summarized using frequency. Pearson *χ*^2^ and *t*-tests were used to compare variables across groups, and corresponding 95% confidence intervals (CIs) were calculated. A *P* value < 0.05 was considered significant for all tests. Multivariate logistic regression models were used to estimate adjusted odds ratios (aORs) and 95% CIs of independent and interactive associations of PA and ST with sleep quality, and physiological, psychological, and social suboptimal health status, after adjusting for age group, gender, student type, residential background, alcohol usage, tobacco usage, and losing weight which these covariates may affect the independent variables. Simple slopes tests were conducted to investigate the interactive associations of PA and ST with poor sleep quality, physiological, psychological, and social suboptimal health status.

It should be noted that this is a more specific continuation of our previous study [2]. Though we used the same database, this study is significantly different from the previous study, with respect to research methods and content. First, we used four response variables as described above. Furthermore, suboptimal health status was divided into three dimensions, instead of viewed as a single dimension. Second, the focus of this study was the independent and interactive associations of ST and PA. Third, PA and ST were viewed as dichotomous variables instead of continuous variables.

### Ethical statement

At the beginning of each survey, the interviewer explained the nature of the survey to participants. Each respondent was asked to provide signed informed consent. Respondents were not included if they refused to participate, which may lead to selection bias. Each participant completed the questionnaire within 30 min. All data were kept strictly confidential. This study was approved by an ethics review committee at Renmin University of China. All methods were performed in accordance with relevant guidelines and regulations [43].

### Patient and public involvement

The study was designed to investigate the associations of PA and ST with physiological, psychological, and social health, especially regarding the effects on sleep quality, among Chinese college students. No patients or members of the public were included in the design, recruitment, or conduct of the study. Measurement results will be disseminated to participants and the public after the study is completed.

## Results

### Demographic characteristics

Participants’ characteristics, stratified by gender, are described in Table 1. We noted some “discrepancies” in sample sizes, which were caused by missing data. Among the 5,233 participants, 3,400 were female (64.97%). The mean age of male participants was significantly higher than that of female participants (22.27 vs. 21.46 years). Most students were bachelor’s or master’s candidates, and most came from urban areas. The proportion of male participants from rural areas was significantly higher than that of female participants. The proportion of high ST among male participants was lower than among female participants (41.41% vs. 47.02%). Moreover, female participants had a significantly lower prevalence of high PA (27.23% vs. 40.35%). The overall prevalence of poor sleep quality was 37.94%, and the difference between male and female participants was not significant. The prevalence rates of physiological, psychological, and social suboptimal health status were 10.43%, 13.18%, and 13.26%, respectively. Compared to male participants, female participants had a significantly higher prevalence of physiological suboptimal health status. Male participants had a higher prevalence of social suboptimal health status.

**Table 1:** Characteristics of surveyed students.

	Total (*n* = 5,233)	Male (*n* = 1,819)	Female (*n* = 3,400)	*P* value
**Age in years (mean±SD)**	21.74±3.58	22.27±3.95	21.46±3.33	<0.001
**Age group**				<0.001
<20	2238 (43.65)	732 (41.22)	1506 (44.94)	
20–25	2223 (43.36)	713 (40.15)	1510 (45.06)	
>25	666 (12.99)	313 (18.64)	335 (10.00)	
**Student type**				<0.001
Bachelor’s	2316 (44.38)	774 (42.25)	1542 (45.35)	
Master’s	2422 (46.41)	818 (44.97)	1604 (47.18)	
Doctoral	481 (9.22)	227 (12.48)	254 (7.47)	
**Residential background**				0.001
Rural	1337 (25.77)	513 (28.37)	824 (24.38)	
Urban	3851 (74.23)	1295 (71.63)	2556 (75.62)	
**Usage of Tobacco**				
Yes	207 (4.0)	183(1.0)	23(0.7)	<0.001
No	5,022 (96.0)	1,632(99.0)	3,377(99.3)	
**Usage of Alcohol**				
Yes	2,334 (44.6)	1,283(71.0)	1,041(30.8)	<0.001
No	2,894 (55.4)	535(29.0)	2,355(69.2)	
**Weight loss**				<0.001
Yes	2481 (47.67)	644 (35.48)	1837 (54.20)	
No	2723 (52.33)	1171 (64.52)	1552 (45.80)	
**ST**				<0.001
Low	2862 (54.93)	1064 (58.59)	1798 (52.98)	
High	2348 (45.07)	752 (41.41)	1596 (47.02)	
**PA**				<0.001
Low	3538 (68.14)	1077 (59.65)	2461 (72.77)	
High	1654 (31.86)	733 (40.35)	921 (27.23)	
**Sleep quality**				0.976
Poor	1194 (37.94)	431 (37.91)	763 (37.96)	
Normal	1953 (62.06)	706 (62.09)	1247 (62.04)	
**Physiological health status**				0.010
SHS	542 (10.43)	162 (8.91)	380 (11.18)	
Healthy	4677 (89.57)	1657 (91.09)	3020 (88.82)	
**Psychological health status**				0.450
SHS	688 (13.18)	231 (12.70)	457 (13.44)	
Healthy	4531 (86.82)	1588 (87.30)	2943 (86.56)	
**Social health status**				<0.001
SHS	692 (13.26)	312 (17.15)	380 (11.18)	
Healthy	4527 (86.74)	1507 (82.85)	3020 (88.82)	

Values are presented as mean±SD or count (percentage). SD: standard deviation; ST: screen time; PA: physical activity; SHS: suboptimal health status. Numbers of missing: age: 106; student type: 14; residential background: 45; weight loss: 29; usage of tobacco: 4; usage of alcohol: 5; ST: 23; PA: 41; sleep quality: 2,086; health status: 14.

### Independent associations of PA and ST with sleep quality and health status

Table 2 shows the independent associations of PA and ST with sleep quality and physiological, psychological, and social suboptimal health status. After adjusting for potential confounders, compared to low PA, high PA was independently associated with a significantly lower risk of poor sleep quality (aOR = 0.64, 95% CI: 0.55–0.76), physiological suboptimal health status (aOR = 0.55, 95% CI: 0.45–0.71), psychological suboptimal health status (aOR = 0.57, 95% CI: 0.47–0.69), and social suboptimal health status (aOR = 0.63, 95% CI: 0.52–0.77). After adjusting for potential confounders, compared to low ST, high ST was independently associated with a significantly higher risk of poor sleep quality (aOR = 1.20, 95% CI: 1.03–1.39), physiological suboptimal health status (aOR = 1.39, 95% CI: 1.16–1.68), psychological suboptimal health status (aOR = 1.43, 95% CI: 1.21–1.69), and social suboptimal health status (aOR = 1.27, 95% CI: 1.08–1.50).

**Table 2:** Associations between ST, PA and sleep quality, and physiological, psychological, and social suboptimal health status.

	Poor sleep quality			Physiological suboptimal health status			Psychological suboptimal health status			Social suboptimal health status		
	n	Crude OR	Adjusted OR^a^	n	Crude OR	Adjusted OR	n	Crude OR	Adjusted OR	n	Crude OR	Adjusted OR
		95%CI	95%CI		95%CI	95%CI		95%CI	95%CI		95%CI	95%CI
**ST**												
Low	621	Ref.	Ref.	254	Ref.	Ref.	324	Ref.	Ref.	354	Ref.	Ref.
High	576	1.22	1.20	289	1.43	1.39	367	1.44	1.43	341	1.20	1.27
		1.05–1.40	1.03–1.39		1.20–1.70	1.16–1.68		1.23–1.69	1.21–1.69		1.02–1.40	1.08–1.50
**PA**												
Low	851	Ref.	Ref.	422	Ref.	Ref.	537	Ref.	Ref.	519	Ref.	Ref.
High	339	0.66	0.64	118	0.58	0.55	154	0.66	0.57	173	0.61	0.63
		0.57–0.77	0.55–0.76		0.50–0.67	0.45–0.71		0.58–0.75	0.47–0.69		0.53–0.70	0.52–0.77

^a^: Adjusting for age group, gender, student type, residential background, alcohol usage, tobacco usage, and losing weight. ST: screen time; PA: physical activity; OR: odds ratio; CI: Confidence interval.

### Interactive associations of ST and PA with sleep quality and health status

The interactive associations of ST and PA with sleep quality and physiological, psychological, and social suboptimal health status are presented in Table 3. We observed significant additive interactive associations of PA and ST with poor sleep quality (*P* < 0.001), physiological suboptimal health status (*P* = 0.001), psychological suboptimal health status (*P* = 0.016), and social suboptimal health status (*P* < 0.001). Compared to those with low PA and high ST, students with high PA and low ST showed significantly lowest risks of poor sleep quality (aOR = 0.56, 95% CI: 0.45–0.70), physiological suboptimal health status (aOR = 0.49, 95% CI: 0.39–0.60), psychological suboptimal health status (aOR = 0.48, 95% CI: 0.39–0.58), and social suboptimal health status (aOR = 0.49, 95% CI: 0.40–0.59). The risks of poor sleep quality (aOR = 0.83, 95% CI: 0.70–0.99), physiological suboptimal health status (aOR = 0.78, 95% CI: 0.67–0.92), psychological suboptimal health status (aOR = 0.74, 95% CI: 0.64–0.86), and social suboptimal health status (aOR = 0.82, 95% CI: 0.70–0.94) were significantly lower in those with low PA and low ST compared with those with low PA and high ST.

**Table 3:** Interactive associations between ST, PA and sleep quality, and physiological, psychological, and social suboptimal health status.

	Low PA			High PA		
	n	Crude OR	Adjusted OR^a^	n	Crude OR	Adjusted OR
		95% CI	95% CI		95% CI	95% CI
**Poor sleep quality**						
High ST	424	Ref.	Ref.	148	0.82 (0.69–0.98)	0.61 (0.48–0.78)
Low ST	427	0.65 (0.51–0.81)	0.83 (0.70–0.99)	191	0.57 (0.46–0.70)	0.56 (0.45–0.70)
**physiological suboptimal health status**						
High ST	469	Ref.	Ref.	129	0.75 (0.65–0.88)	0.62 (0.49–0.78)
Low ST	428	0.58 (0.47–0.73)	0.78 (0.67–0.92)	145	0.45 (0.37–0.55)	0.49 (0.39–0.60)
**psychological suboptimal health status**						
High ST	591	Ref.	Ref.	192	0.72 (0.63–0.83)	0.70 (0.57–0.86)
Low ST	538	0.71 (0.58–0.86)	0.74 (0.64–0.86)	202	0.47 (0.39–0.56)	0.48 (0.39–0.58)
**Social suboptimal health status**						
High ST	574	Ref.	Ref.	167	0.82 (0.74–0.98)	0.58 (0.47–0.71)
Low ST	576	0.62 (0.51–0.76)	0.82 (0.70–0.94)	212	0.52 (0.43–0.63)	0.49 (0.40–0.59)

^a^: Adjusting for age group, gender, student type, residential background, alcohol usage, tobacco usage, and losing weight. ST: screen time; PA: physical activity; OR: odds ratio; CI: Confidence interval.

In addition, the odds ratios of associations were smaller when students reported high PA and low ST than those with low PA and low ST, respectively. Compared to their counterparts, the subjects with high PA and low ST had 51% lower odds of physiological l suboptimal health status, whereas within the low PA group, subjects with low ST were 22% less likely to physiological suboptimal health status than their peers. Compared to their partners, the subjects with high PA and low ST had 52% lower odds of psychological suboptimal health status, whereas within the low PA group, subjects with low ST were 26% less likely to psychological suboptimal health status than their peers. The students with high PA and low ST had 51% lower odds of social suboptimal health status, whereas within the low PA group, subjects with low ST were 18% less likely to social suboptimal health status than their peers. Compared to their counterparts, the students with high PA and low ST had 44% lower odds of poor sleep quality, whereas within the low PA group, subjects with low ST were 17% less likely to poor sleep quality than their peers. These results show that PA and ST interact and have an additive effect on physiological health, psychological health, social health, and sleep quality.

**Fig 1:**
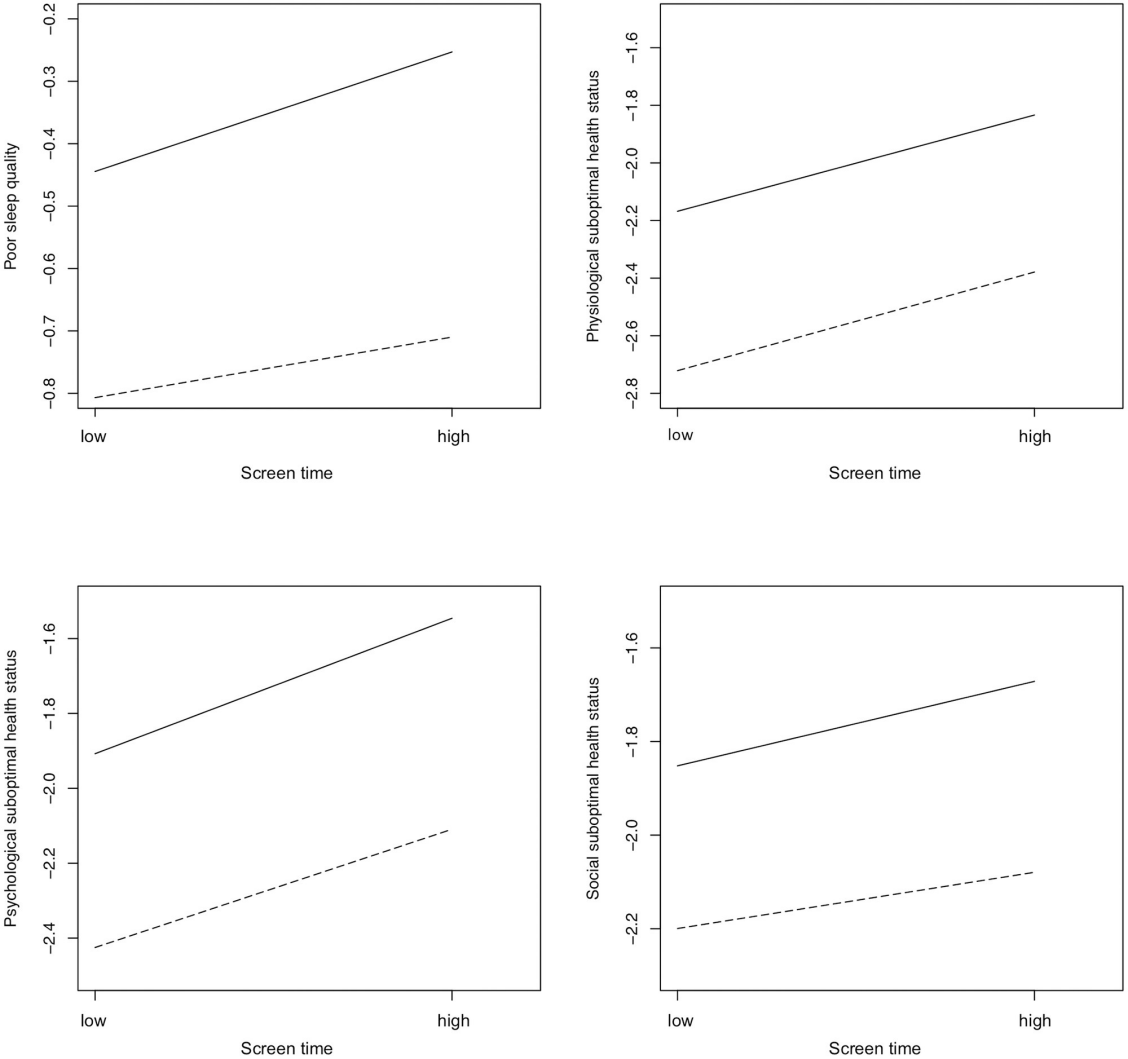
Interactive associations of ST and PA with sleep quality, and physiological, psychological, and social suboptimal health status. Note: Solid line: low PA; dashed line: high PA. Y axis is the logarithm of aOR.

The interactive associations of ST and PA with sleep quality and physiological, psychological, and social suboptimal health status are graphically presented in Fig 1. The results of simple slopes tests suggested interactive associations of PA and ST with poor sleep quality (*P* = 0.013), physiological suboptimal health status (*P* < 0.001), psychological suboptimal health status (*P* < 0.001), and social suboptimal health status (*P* = 0.024).

## Discussion

The findings of the present study suggested that low PA and high ST are independently associated with self-reported poor sleep quality, as well as physiological, psychological, and social suboptimal health status among Chinese college students. Additionally, interactive associations between low PA and high ST with poor sleep quality, as well as physiological, psychological, and social suboptimal health status, were also observed.

### Suboptimal health status

Our analysis suggested that high PA can have a positive association with physiological health among Chinese college students. Some published studies also reported associations between PA and physiological health [25, 44, 45]. The benefits of PA on health are well documented. A research has reported that regular PA can lead to lower blood pressure in individuals with hypertension, as shown in 27 randomized controlled studies [46]. Some studies reported that PA was associated with multiple health benefits for college students, such as lower blood cholesterol, lower blood pressure, reduced incidence of metabolic syndromes, reduced obesity, and higher bone density [24, 47–49]. Our analysis further suggested that low PA is associated with psychological suboptimal health status. Several published studies suggested that regular PA can promote mental health in various ways, including improved self-esteem, self-efficacy, cognitive function, and psychological function and decreased distress [50–52]. A meta-analytic study (including 92 studies with a total of 4,310 participants with depression and 306 studies with a total of 10,755 participants with anxiety) reported that PA reduces depression and anxiety in nonclinical populations [53]. Increased PA has also been associated with significant reductions in depression, anxiety, and suicidal ideation among college students [54, 55]. One possible mechanism for this could be that PA can promote brain development and improve cognitive function in young adults [56]. Another potential reason is an increase in serotonin or other neurotransmitters associated with endorphins, generated as a result of PA [25]. An association between PA and social health status was observed in the present study. In addition to better physical health, PA was observed to be associated with better social health (e.g., self-image, quality of life, family and peer relationships). A previous study also reported that, compared to other forms of PA, team-based PA is associated with better social health outcomes [57]. A possible explanation is that regular PA helps relieve stress, enhance self-confidence, extend one’s social circle, and establish good interpersonal relationships [34].

The associations of high ST with physiological, psychological, and social health have been studied since the onset of popularity of electronic devices [58]. Our analysis suggested that low ST and high PA were negative associated with physiological suboptimal health status independently, as well as synergistically, in college freshmen. One theory for such negative association is that ST displaces time available for PA [59]. Our results also suggested that low ST and high PA have negative association with psychological suboptimal health status, independently and interactively, among Chinese college freshmen. The adverse association of high ST with psychological health has also been reported in several previous studies. A self-reported study on 4,747 college students demonstrated that ST may represent a risk factor or marker for anxiety, depression and psychopathological symptoms [30]. Associations between reduced psychological wellbeing, lower PA levels and increased ST in adolescents have been reported [60]. Our results extended the current literature by examining the interactive association of PA and ST with psychological health among college students. Compared to those with high ST, significantly lower risks of psychological suboptimal health status in participants with low ST were found in the low and high PA groups. In addition, the odds ratios of associations were smaller when students reported high PA and low ST than those with low PA and low ST. This result shows that PA and ST interact and have an additive effect on psychological health. The mechanisms for adverse health effects are complex, yet one possible explanation is that spending more time sitting in front of a television or computer can increase metabolic risk [61], which is associated with poor mental health [62]. Another possibility is that high ST may cause gray matter and white matter abnormalities in the brain [63]. Some researchers explained the negative effects of ST on mental health by the content of television programs and computer games [52].

We had similar findings regarding the negative association of high ST on social health. Our results suggested that low ST and high PA were negative related to social suboptimal health status, independently and synergistically, in college freshmen. As mentioned above, previous studies confirmed a negative association between high ST and mental health. Depression, anxiety, low self-esteem, low self-efficacy, cognitive impairments and other psychological problems may greatly affect life, work, study and interpersonal interactions, leading to bad social experiences and even antisocial. Another possible reason for this negative relationship between high ST and social health is that students may spend less time developing social skills if they spend more time using electronic devices. Furthermore, students may reduce their amount of physical activity, which has been shown to impair social ability [64].

### Sleep quality

Poor sleep quality is highly prevalent among college students. For example, in the Williams’ study, 51.8% of 3,461 Chilean college students exhibited poor sleep quality [65]. Some studies have further suggested that high ST harms sleep quality among school-aged children and adolescents [66]. In the present study, we found the prevalence of poor sleep quality among Chinese college freshmen to be 37.79%, and low ST and high PA were positive related to poor sleep quality, both independently and interactively. Compared to those with high ST, significantly lower risks of poor sleep quality in participants with low ST were found in both the low and high PA groups. In addition, the odds ratios of associations were smaller when students reported high PA and low ST than those with low PA and low ST. High ST has been believed to be a cause of insufficient and low-quality sleep, which may be explained by time displacement [67]. When more time is spent in front of a screen, less time is available for sleeping. Moreover, it has also been suggested that longer screen time may affect sleep by reducing the time spent doing other activities, such as exercise, which may benefit sleep quality and sleep regulation [68]. Thus, sleep and PA influence each other through complex, bilateral interactions that involve multiple physiological and psychological pathways [69]. Some studies have reported that regular PA can benefit sleep and potentially cure sleep disorders [70]. Previous studies have also found that PA can increase slow wave sleep in children, which may benefit sleep regulation [71]. Additionally, when taking the associations of PA with physiological and psychological health into consideration, there is a good possibility that PA promotes sleep quality by improving mental well-being [42]. However, the mechanisms underlying this relationship require further investigation. Although there are still uncertainties, evidence found in this study, along with that of previous literature, show a favorable association of limiting ST and increasing PA with sleep quality.

This study inevitably had limitations. Due to the cross-sectional design and observational nature of this study, we cannot infer causal relationships. Furthermore, because PA and ST were self-reported, neither recall nor reporting bias can be ruled out. Our study only measured the frequencies of ST and PA, but did not assess the types of screen-based activities, duration and intensity of PA. Therefore, the associations maybe more complicated. The presence of poor sleep quality and physiological, psychological, and social suboptimal health status were assessed using standardized questionnaires, which may not be as accurate as clinical diagnoses. Therefore, additional studies with clinically diagnostic interviews may be needed. The information collected may also be limited. This study adjusted for confounders such as age, gender, student type, residential background, alcohol use, tobacco use, and weight loss; however, there are other potential confounders, such as drug abuse and caffeine consumption. Furthermore, some covariates, such as alcohol use and tobacco use, were considered as dichotomy which is not sufficiently informative. Finally, our results may not represent all Chinese young adults, as the participants came only from one large university.

In conclusion, our findings indicated that low ST and high PA are independently and interactively associated with physiological, psychological, and social suboptimal health status, as well as poor sleep quality, among Chinese college freshmen. Our findings further supported that increasing PA and reducing ST should be included when planning health promotion strategies among young people. Future research should aim to measure the impact of interventions and potential consequences on overall health and sleep quality.

## Supporting information

S1 File English copy of the questionnaire. (DOCX)

S2 File The questionnaire of Pittsburgh Sleep Quality Index (PSQI). (PDF)
