# Supplementary material for: Associations of physical activity and screen time with suboptimal health status and sleep quality among Chinese college freshmen: A cross-sectional study
Source: PLoS One. 2020 Sep 18;15(9):e0239429. doi: 10.1371/journal.pone.0239429 (PMC7500622; doi:10.1371/journal.pone.0239429)
Supplement: S2 File — (PDF) [file pone.0239429.s002.pdf]

Name________________________________________________________________ Date_________

# Sleep Quality Assessment (PSQI)

## What is PSQI, and what is it measuring?

The Pittsburgh Sleep Quality Index (PSQI) is an effective instrument used to measure the quality and patterns of sleep in adults. It differentiates "poor" from "good" sleep quality by measuring seven areas (components): subjective sleep quality, sleep latency, sleep duration, habitual sleep efficiency, sleep disturbances, use of sleeping medications, and daytime dysfunction over the last month.

# INSTRUCTIONS:

The following questions relate to your usual sleep habits during the past month only. Your answers should indicate the most accurate reply for the majority of days and nights in the past month. Please answer all questions.

## During the past month,

1. When have you usually gone to bed? ______________________________
2. How long (in minutes) has it taken you to fall asleep each night? ______________________________
3. What time have you usually gotten up in the morning? ______________________________
4. A. How many hours of actual sleep did you get at night? ______________________________
   B. How many hours were you in bed? ______________________________

| 5. During the past month, how often have you had trouble sleeping because you | Not during the past month (0) | Less than once a week (1) | Once or twice a week (2) | Three or more times a week (3) |
|---|---|---|---|---|
| A. Cannot get to sleep within 30 minutes | | | | |
| B. Wake up in the middle of the night or early morning | | | | |
| C. Have to get up to use the bathroom | | | | |
| D. Cannot breathe comfortably | | | | |
| E. Cough or snore loudly | | | | |
| F. Feel too cold | | | | |
| G. Feel too hot | | | | |
| H. Have bad dreams | | | | |
| I. Have pain | | | | |
| J. Other reason (s), please describe, including how often you have had trouble sleeping because of this reason (s): | | | | |
| 6. During the past month, how often have you taken medicine (prescribed or "over the counter") to help you sleep? | | | | |
| 7. During the past month, how often have you had trouble staying awake while driving, eating meals, or engaging in social activity? | | | | |
| 8. During the past month, how much of a problem has it been for you to keep up enthusiasm to get things done? | | | | |
| 9. During the past month, how would you rate your sleep quality overall? | Very good (0) | Fairly good (1) | Fairly bad (2) | Very bad (3) |

## Scoring

| | | |
|---|---|---|
| **Component 1** | #9 Score | C1 ___________ |
| **Component 2** | #2 Score (<15min (0), 16-30min (1), 31-60 min (2), >60min (3))<br>+ #5a Score (if sum is equal 0=0; 1-2=1; 3-4=2; 5-6=3) | C2 ___________ |
| **Component 3** | #4 Score (>7(0), 6-7 (1), 5-6 (2), <5 (3) | C3 ___________ |
| **Component 4** | (total # of hours asleep) / (total # of hours in bed) x 100<br>>85%=0, 75%-84%=!, 65%-74%=2, <65%=3 | C4 ___________ |
| **Component 5** | # sum of scores 5b to 5j (0=0; 1-9=1; 10-18=2; 19-27=3) | C5 ___________ |
| **Component 6** | #6 Score | C6 ___________ |
| **Component 7** | #7 Score + #8 score (0=0; 1-2=1; 3-4=2; 5-6=3) | C7 ___________ |

*Add the seven component scores together* __________________ *Global PSQI* _______________

***A total score of "5" or greater is indicative of poor sleep quality.***

***If you scored "5" or more it is suggested that you discuss your sleep habits with a healthcare provider***
